# Multi-omics analyses reveal new insights into nutritional quality changes of alfalfa leaves during the flowering period

**DOI:** 10.3389/fpls.2022.995031

**Published:** 2022-11-30

**Authors:** Yinghao Liu, Wenqiang Fan, Qiming Cheng, Lianyi Zhang, Ting Cai, Quan Shi, Zuo Wang, Chun Chang, Qiang Yin, Xiaowei Jiang, Ke Jin

**Affiliations:** ^1^ Key Laboratory of Forage Cultivation, Processing and High Efficient Utilization of Ministry of Agriculture and Rural Affairs, Institute of Grassland Research of Chinese Academy of Agricultural Sciences, Hohhot, China; ^2^ Key Laboratory of Grassland Resources, Ministry of Education, Inner Mongolia Agricultural University, Hohhot, China; ^3^ College of Animal Science, Guizhou University, Guiyang, China; ^4^ Key Laboratory of Efficient Utilization of Forage, Inner Mongolia Agricultural and Animal Husbandry Technology Extension Center, Hohhot, China

**Keywords:** alfalfa leaves, metabolomics, proteomics, transcriptomics, chlorophyll, amino acids, flavonoids

## Abstract

High-quality alfalfa is an indispensable resource for animal husbandry and sustainable development. Its nutritional quality changes dramatically during its life cycle and, at present, no molecular mechanisms for nutrient metabolic variation in alfalfa leaves at different growth stages have been clearly reported. We have used correlation and network analyses of the alfalfa leaf metabolome, proteome, and transcriptome to explore chlorophyll, flavonoid, and amino acid content at two development stages: budding stage (BS) and full-bloom stage (FBS). A high correlation between the expression of biosynthetic genes and their metabolites revealed significant reductions in metabolite content as the plant matured from BS to FBS. l-Glutamate, the first molecule of chlorophyll biosynthesis, decreased, and the expression of *HemA*, which controls the transformation of glutamyl-tRNA to glutamate 1-semialdehyde, was down-regulated, leading to a reduction in leaf chlorophyll content. Flavonoids also decreased, driven at least in part by increased expression of the gene encoding CYP75B1: flavonoid 3′-monooxygenase, which catalyzes the hydroxylation of dihydroflavonols and flavonols, resulting in degradation of flavonoids. Expression of *NITRILASE 2 (NIT2)* and *Methyltransferase B* (*metB)*, which regulate amino acid metabolism and influence the expression of genes of the glycolysis-TCA pathway, were down-regulated, causing amino acid content in alfalfa leaves to decrease at FBS. This study provides new insights into the complex regulatory network governing the content and decrease of chlorophyll, amino acids, flavonoids, and other nutrients in alfalfa leaves during maturation. These results further provide a theoretical basis for the generation of alfalfa varieties exhibiting higher nutritional quality, high-yield cultivation, and a timely harvest.

## Introduction

Alfalfa (*Medicago sativa* L.) is a high-yield perennial forage legume with excellent nutrition, digestibility, and palatability. It is a good source of feed for high-yielding dairy cows as it is rich in protein, amino acids, chlorophyll, flavonoids, and other nutritious substances (Popovic et al., 2001). Metabolic processes occurring in the alfalfa leaf, such as photosynthesis, respiration, and transpiration, are closely related to plant growth, nutrition, yield, quality, and resistance (Dong et al., 2021). Therefore, study of alfalfa leaves at different growth stages may provide information to ensure high yield and nutrient quality.

To date, only changes in alfalfa leaf protein metabolism have been studied at different developmental stages (Fan et al., 2018); there are few reports on how chlorophyll, amino acid, and flavonoid metabolism change with development. Chlorophyll is indispensable for absorption and transduction of light energy during photosynthesis (Gao et al., 2018). Chlorophyll biosynthesis is a tightly regulated process, since its derivatives can produce highly toxic compounds upon illumination if not bound to specific proteins, such as HEMA1 and chlorophyll synthase ChlG (Stenbaek and Jensen, 1995). Overexpression in tobacco of HEMA1, which exhibits glutamyl-tRNA reductase activity, has been shown to increase chlorophyll content and the rate of photosynthesis (Zhang et al., 2010; Schmied et al., 2011). ChlG regulates the chlorophyll biosynthetic pathway by modulating stable assembly of chlorophyll-binding proteins and other thylakoid membrane components (Shalygo et al., 2009). Mutations in genes involved in the chlorophyll synthetic pathway can cause alterations in chlorophyll content and leaf color. For example, in rice, mutation in the gene encoding protoporphyrin IX methyltransferase, which catalyzes formation of protoporphyrin IX mono-methyl ester, results a yellow-green plant leaf phenotype (Wang et al., 2017).

Flavonoids are widely distributed plant secondary metabolites. These polyphenolic compounds are synthesized *via* the phenylpropanoid pathway and classified into ten major group: flavones, isoflavones, flavanones, flavonols, dihydroflavonols, flavan-3-ols (F3Os), anthocyanins, anthocyanidins, leucoanthocyanidins (flavan-3,4-diols), and polymeric proanthocyanidins (PAs) (Dantas et al., 2020). The most common alfalfa flavonoids are glycosides of the flavone aglycones apigenin, luteolin, tricin, and chrysoeriol (Goawska et al., 2010). In animals, the flavonoids in alfalfa mainly have biological activities such as anti-oxidation, anti-aging, anti-tumor and enhancing body immunity (Jing et al., 2015). Thus, alfalfa flavonoids can significantly improve the production performance and antioxidant capacity in animals, such as cattle (Zhan et al., 2017) and broiler chickens (Ouyang et al., 2016).

A third group of important metabolites are amino acids, which are the main circulating form of plant nitrogen. Amino acid metabolism through the tricarboxylic acid (TCA) cycle generates energy and essential elements for protein synthesis, carbohydrate metabolism, endogenous hormone regulation, and energy storage (Less and Galili, 2009). Although legumes have a high content of organic nitrogen, they, like other plants, contains low levels of some essential amino acids — lysine (Lys), methionine (Met), threonine (Thr), and tryptophan (Trp) — constraining their nutritional quality for animals, including humans (Galili et al., 2016). Cysteine and methionine can also be limiting, particularly for ruminants; in cattle, methionine increases both yield and protein content in milk (Bird and Moir, 1972; Tong et al., 2014).

Plant leaf nutrient absorption is one of the important processes in nutrient utilization and circulation. Previous studies have shown that leaf nutrients are affected by temporal and spatial environmental factors (temperature, precipitation, habitat, etc.), and their responses often show different patterns within and between species. For some species (e.g., alfalfa), the uptake and utilization of nutrients vary with growth (growth period, stubble, age change), leading to changes in plant nutrient reuptake. So questions arise, such as what is the performance of nutrient reuptake in the leaves of perennial forage legumes at different growth stages? And what is its mechanism of regulation?

To date, few studies have linked gene and protein expression with molecular mechanisms that regulate metabolite accumulation and nutritional quality in alfalfa leaves during the flowering period. Here, we have combined transcriptomics, proteomics, and broad metabolomics analyses to explore accumulation of primary and secondary metabolites in alfalfa leaves at different developmental stages, focusing on chlorophyll, flavonoid, and amino acid metabolism. Our results will provide new insights and avenues to improve alfalfa leaf nutritional quality, particularly during flowering.

## Materials and methods

### Plant material and sampling

Alfalfa plants (*Medicago sativa* L. var. WL319HQ) were planted in 2019 and assessed during the 2020 growing season in the experimental field of the Inner Mongolia Agricultural University (Hohhot, Inner Mongolia, China). Leaf samples were collected at two different development stages, namely the budding stage (BS, lower buds appeared in >80% of the branches) and full-bloom stage (FBS, 80% flowering). On June 1, 2020 (BS), 60 plants at a uniform growth stage were selected. Leaves from half of these plants (30) were collected and mixed to generate the BS samples. The remaining 30 plants were allowed to grow naturally for 2 weeks (15 June), when leaves were harvested to generate the FBS samples. Leaves from 10 plants were combined at each stage to generate the three biological replicates used for subsequent analyses.

Leaf sampling occurred in the same way for BS and FBS stages. All leaves from the above ground part of the plant were harvested and mixed to provide an accurate assessment of whole-plant nutritional content as delivered for animal forage. Leaves were harvested at 6pm, when light intensity and temperature are relatively low; at this time, plant metabolism is relatively slow, so this time was chosen to minimize individual plant differences in metabolism. Samples were immediately flash-frozen in liquid nitrogen and stored at -80°C until analysis.

### Chemical composition analysis

Amino acid content was determined according to the national standard of the People’s Republic of China, GB/T 18246-2000 “Determination of Amino acids in Feed” (Dong et al., 2018). Flavonoid content was determined by colorimetry (Guo et al., 2020); and chlorophyll content by spectrophotometry (Harmut, 1987).

### Untargeted metabolomics analysis

Freeze-dried samples were homogenized into powder. Metabolites were extracted from approximately 80 mg of sample in 1.2 mL 70% (w/v) aqueous methanol overnight at 4°C. After centrifugation to remove undissolved residue, the supernatant was absorbed and filtered, and stored at -80°C before the analysis. Quality control (QC) samples were prepared by pooling 10 μl of each sample.

UPLC-MS/MS was performed as described by Wang et al. (2019), with a minor modification that the gradient was kept at 40% B for only 2.9 min before final increase to 85% and column re-calibration. to monitor 10 candidate ions per cycle.

Qualitative analysis was performed by searching a self-compiled database (Shanghai Applied Protein Technology Co., Ltd.). Significantly different expressed metabolites (DEMs) were determined by Variable Importance in Projection (VIP) ≥1 and fold-change >1.5 or <0.67 (Wang et al., 2019).

### Tandem mass tag-based proteomic analysis

The freeze-dried samples were ground into a fine powder. Sample lysis, protein extraction, and filter-aided sample preparation (FASP Digestion) were performed as described previously (Wisniewski et al., 2009).

Samples (100 μg peptide) were labeled using a tandem mass tag (TMT) Isobaric Label Reagent Set (Thermo Fisher Scientific Inc., Waltham, MA, USA). A Pierce high pH reversed-phase fractionation kit (Thermo Fisher Scientific Inc., Waltham, MA, USA) was used to fractionate TMT-labeled digest samples into nine fractions according to manufacturer’s instructions. LC-MS/MS analysis were performed as described by Ren et al. (2020).

The MS raw data for each sample were searched, identified, and quantified using the MASCOT engine (Matrix Science, London, UK; version 2.2) embedded into Proteome Discoverer 1.4 software. Differentially expressed proteins (DEPs) were selected using fold-change >1.2 or <0.83 and P-value < 0.05 (Jing et al., 2020).

### Transcriptomic analysis

RNA was extracted, and the RNA-sequencing (RNA-seq) library constructed and sequenced as described by Xu et al. (2022). Six libraries were analyzed (three biological replicates at two developmental stages), using the alfalfa genome from: https://figshare.com/articles/dataset/genome_fasta_sequence_and_annotation_files/12327602. Differentially expressed genes (DEGs) were selected with DESeq2 software using p-adjust<0.05 and |log_2_ fold change|≥1 as described by Xu et al., 2022.

### Bioinformatics analysis

Bioinformatic analyses of DEGs, DEPs and DEMs were performed with Python programming. At p-adjust<0.05, it was considered that Gene Ontology (GO, http://www.geneontology.org/) gene function and KEGG pathways (http://www.genome.jp/kegg/) were significantly enriched. DEGs in the protein–protein interaction (PPI) network were predicted by STRING (https://string-db.org/), and the drawn network diagram and network node degree were analyzed by Cytoscape 3.5.1. Transcription factors (TFs) were identified using PlantTFDB 4.0 (http://planttfdb.cbi.pku.edu.cn/).

### Statistical analysis

Chemical composition data were analyzed by one-way ANOVA.

For metabolomics analyses, multivariate statistical analysis, SIMCA-P (version 14.1, Umetrics, Umea, Sweden) was employed. After Pareto scaling, principal component analysis (PCA) and orthogonal partial least-squares discriminant analyses (OPLS-DA) were performed. The variable importance in projection (VIP) values were generated from the OPLS-DA model.

Analysis of GO and KEGG enrichment was performed using Fisher’s exact test and Benjamini- Hochberg correction for multiple testing was further applied to adjust derived p-values.

The coexpression network analyses was screened by Pearson correlation analysis.

Further comparisons were performed as described in figure legends. All statistical analyses were plotted as described by Wang et al. (2021).

## Results

### Physiological analysis

**Figure 1:**
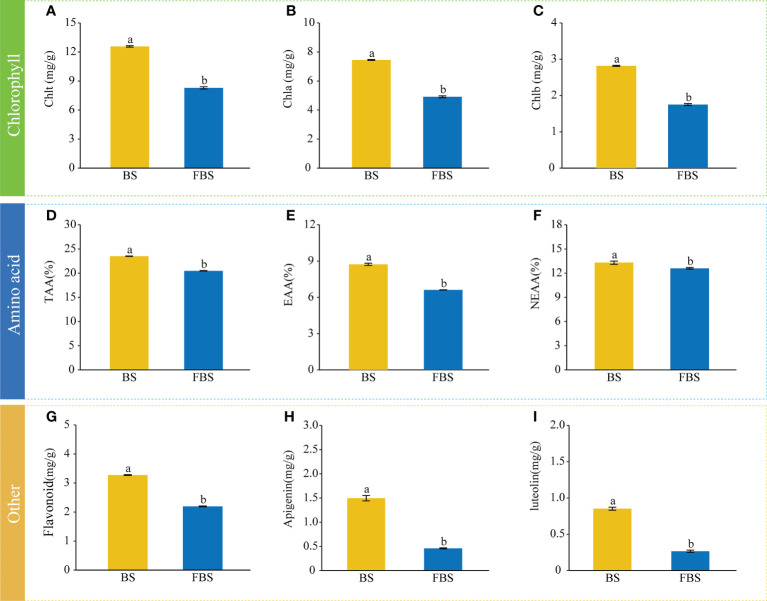
Abundances of metabolites involved in chlorophyll, amino acid, and flavonoid biosynthesis during budding stage (BS) and full-bloom stage (FBS). **(A)** total chlorophyll Chl_t_, **(B)** chlorophyll a Chl_a_, **(C)** chlorophyll b Chl_b_, **(D)** total amino acids TAA, **(E)** essential amino acids EAA, **(F)** non-essential amino acids NEAA, **(G)** flavonoids, **(H)** apigenin, and **(I)** luteolin. Letters indicate significant differences (P<0.05, Duncan’s method).

The chlorophyll, amino acid, and flavonoid content of alfalfa leaves was determined and quantified at different developmental stages (**Figure 1**). For all analyzed compounds, the content was significantly higher (P < 0.05) in the budding stage (BS) than in the full-bloom stage (FBS). The total chlorophyll content was 12.57 mg/g at BS and 8.29 mg/g at FBS, respectively (**Figure 1A**). Consistently, the contents of Chl_a_ and Chl_b_ at BS (7.45 mg/g and 4.91 mg/g, respectively) were higher than at FBS (2.82 and 1.75, respectively) (**Figures 1B, C**). The total amino acid (TAA) content in alfalfa leaves was 23.51% at BS, higher than the 20.49% at FBS (**Figure 1D**). When looking at the essential amino acid (EAA, **Figure 1E**) and nonessential amino acid (NEAA, **Figure 1F**) content, BS leaves contained 8.7% EAA and 13.3% NEAA compared with 6.6% EAA and 12.6% NEAA in FBS leaves. Flavonoid analysis showed that the total flavonoid (**Figure 1G**) content of alfalfa leaves was with 3.27 mg/g at BS, higher than 2.19 mg/g at FBS. This trend was also observed for specific flavonoids, including apigenin (**Figure 1H**, 1.5 mg/g *vs.* 0.46 mg/g) and luteolin (**Figure 1I**, 0.85mg/g *vs.* 0.27 mg/g).

### Untargeted metabolomic analysis

To explore how leaf nutritional quality changes during alfalfa development, the metabolomic composition of BS and FBS leaves was compared (**Supplementary Figure 1A**). Repeatability and reliability of the MS data was verified *via* QC sample test curves (**Supplementary Figure 1B**). 56 metabolites clearly separated into two groups (BS and FBS) by Principal Component Analysis (PCA, **Supplementary Figure 1C**), and further delineated using OPLS-DA to identify differentially abundant metabolites (**Supplementary Figure 1D**). That R^2^ Y and Q^2^ Y scores were both ≥0.8 confirms the correctness of the differences identified in the two growth stages in alfalfa leaf metabolism.

**Figure 2:**
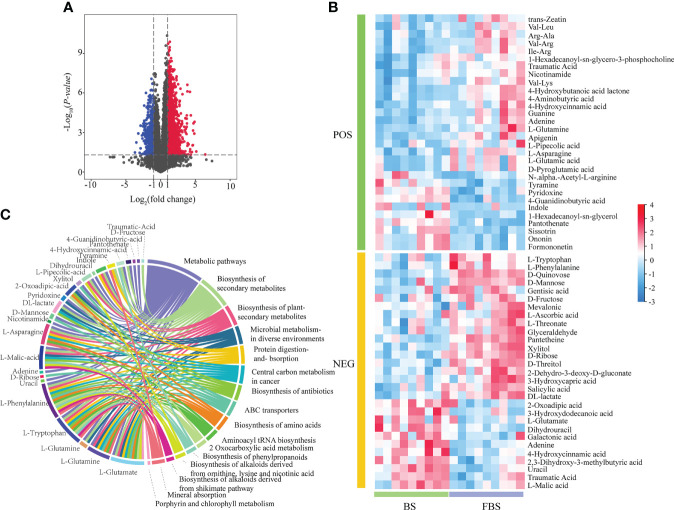
**(A)** Volcano map of differentially expressed metabolites. **(B)** Heat map of differentially expressed metabolites. **(C)** Circle diagram of GO enrichment analysis of differentially expressed metabolites.

Metabolite abundance was quantified based on mass spectrum signal intensity (**Figure 2A**). At FBS, the signal intensity of Chl, amino acids, and flavonoids decreased by 23.46%, 12.36%, and 14.29%, respectively, compared with levels during BS; and 24 and 25 upregulated and downregulated metabolites, respectively, were identified (**Figure 2B**). Differentially expressed metabolites (DEMs) were used for KEGG enrichment analysis. In **Figure 2C**, the left semi-circle shows DEMs, while the right semi-circle shows enriched pathways, with lines linking DEMs with pathways. DEMs were enriched in *Metabolic pathways* and *Biosynthesis of secondary metabolites*, especially pathways involving l-glutamate, l-glutamine, l-tryptophan, and l-phenylalanine (**Figure 2C**).

### TMT-based proteomic analysis

**Figure 3:**
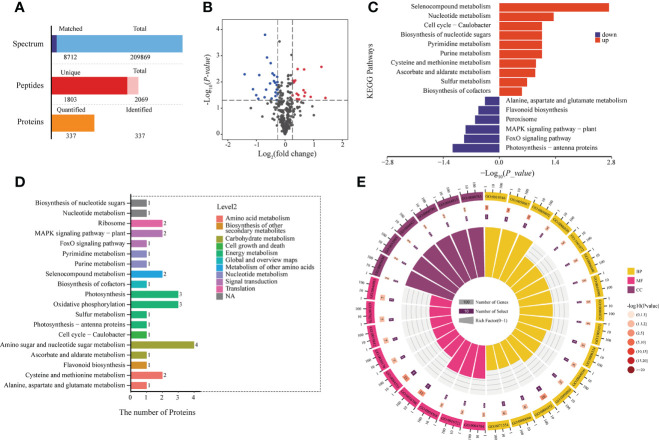
**(A)** Histogram of protein identification and quantitative results. **(B)** Volcano map of differentially expressed proteins. **(C)** KEGG enrichment of up-regulated proteins. **(D)** KEGG results of all differentially expressed proteins. **(E)** GO enrichment of all differentially expressed proteins.

The quality and quantity of plant metabolites depends on the presence of the metabolizing proteins, so we analyzed differential protein expression between the two developmental stages. A total of 337 proteins was identified (**Figure 3A**); 38 proteins showed significant changes in expression from BS to FBS, including 15 upregulated and 23 downregulated proteins (**Figure 3B**).

The proteomics KEGG analysis for metabolism and signaling pathways is presented in **Figure 3C**, and the number of proteins per pathway in **Figure 3D**. In total, 10 pathways were upregulated and six down-regulated. Of the upregulated pathways, the amino sugar and nucleotide sugar metabolism pathway was the most enriched. In line with the metabolite analysis, photosynthesis and flavonoid metabolism pathways were downregulated. To further characterize the differential protein expression, a GO analysis was performed. Enrichment could be found in 15 categories of biological processes (BP), nine in molecular functions (MF), and six in cellular components (CC) (**Figure 3E**).

### Transcriptomic analysis

After filtering low-quality reads, 245,922,094 clean reads were obtained from our six sequenced libraries (**Supplementary Table 1**). Clean reads from each sample were aligned to the alfalfa reference genome, and 96,267 expressed genes were detected.

**Figure 4:**
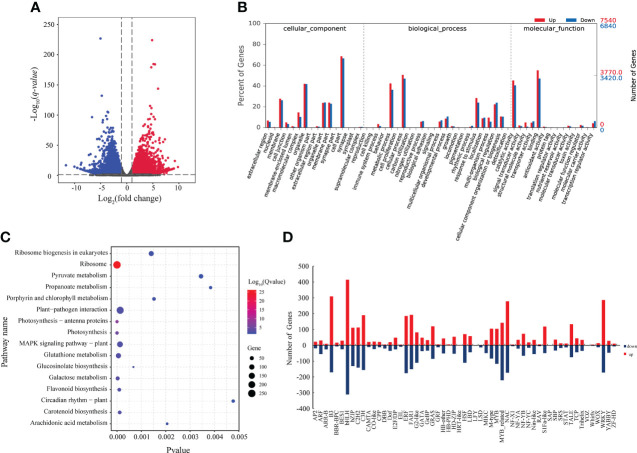
**(A)** Volcano map of DEGs. **(B)** GO enrichment analysis of DEGs. **(C)** KEGG enrichment of DEGs. **(D)** Statistical map of differentially expressed transcription factor families.

Our transcriptomic analysis revealed that 7,537 genes were upregulated, and 6,843 genes were downregulated at FBS compared to BS (**Figure 4A**). To further analyze the function of proteins encoded by DEGs, a GO analysis was performed, and 15 categories were enriched in biological processes (BP), nine in molecular functions (MF), and six in cellular components (CC) (**Figure 4B**). Similar to the metabolomic and proteomic analyses, the KEGG pathway analysis of the transcriptome indicated that 13 genes were differentially expressed involved in Ribosome biogenesis in eukaryotes, two in Ribosome, and one in Photosynthesis (**Figure 4C**).

A total of 7,228 DEGs encoding 26 families of transcription factors were identified (**Figure 4D**), including the large transcription factor families bHLH, MYB, and YABBY. These results indicate that these transcription factors participate in regulating changes in gene expression between BS and FBS.

### Expression patterns of gene and proteins within chlorophyll, amino acids, and flavonoids synthetic pathways

**Figure 5:**
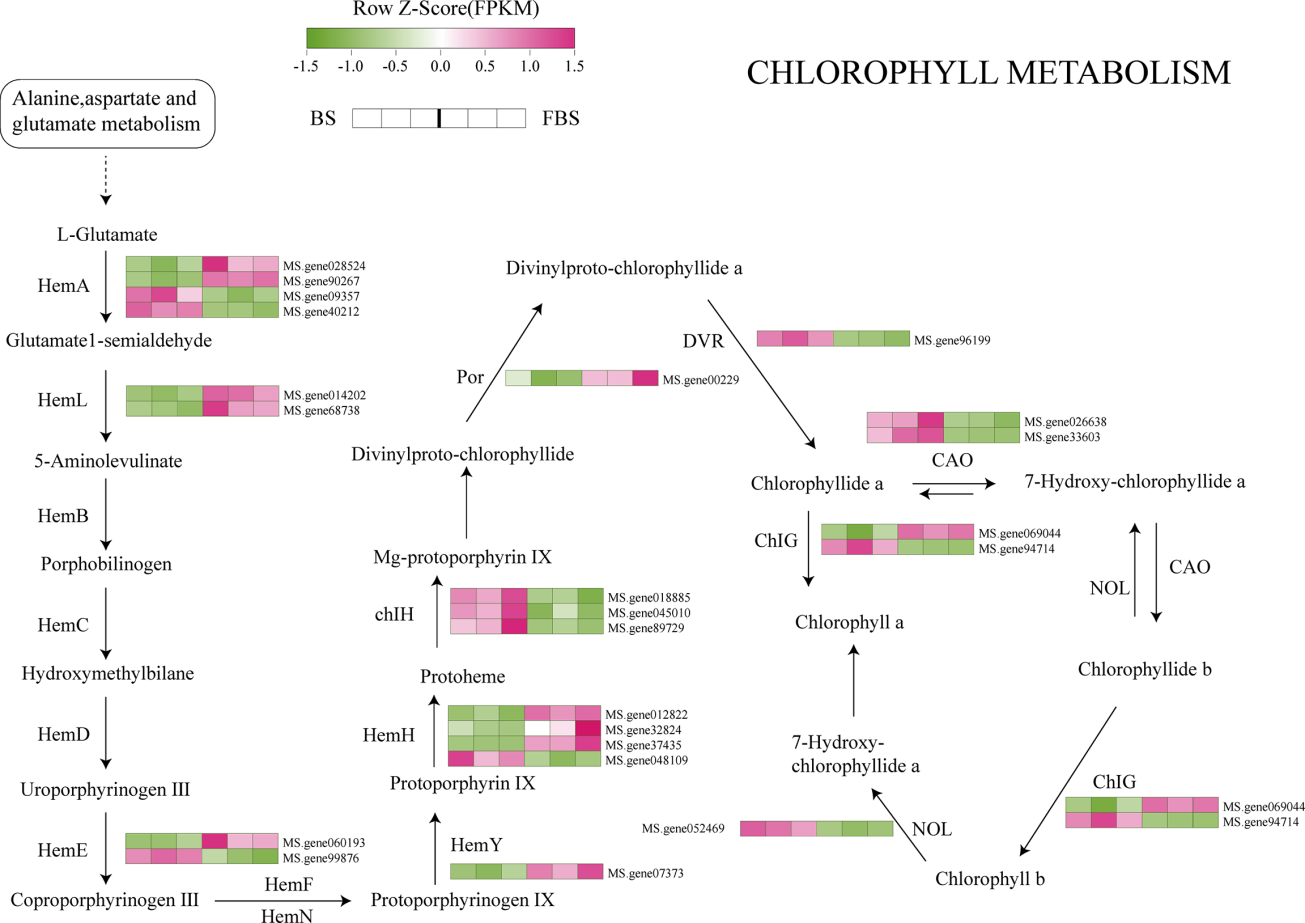
Chlorophyll biosynthesis pathway highlighting differentially expressed genes.

**Figure 6:**
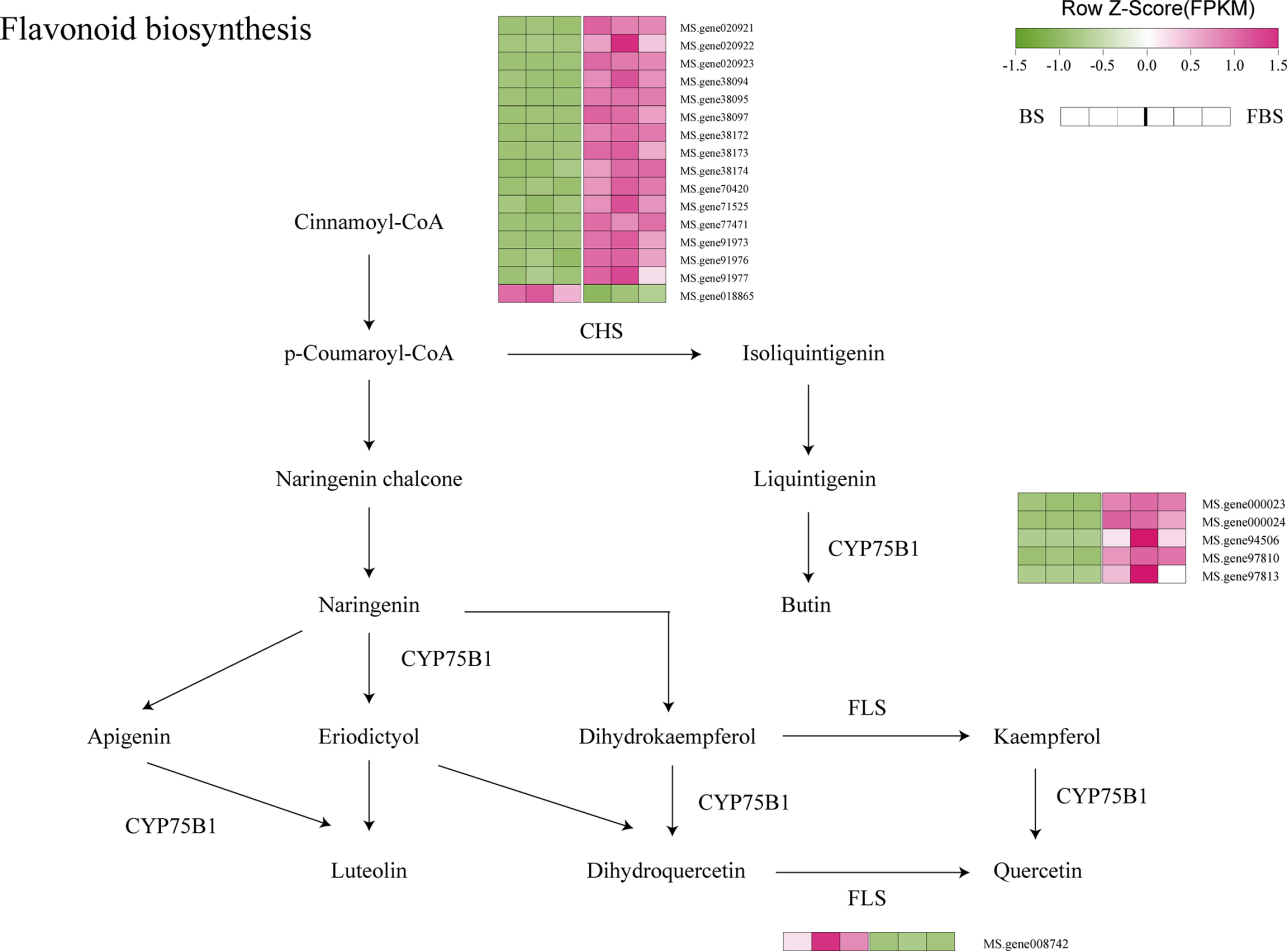
Flavonoid biosynthesis pathway highlighting differentially expressed genes.

**Figure 7:**
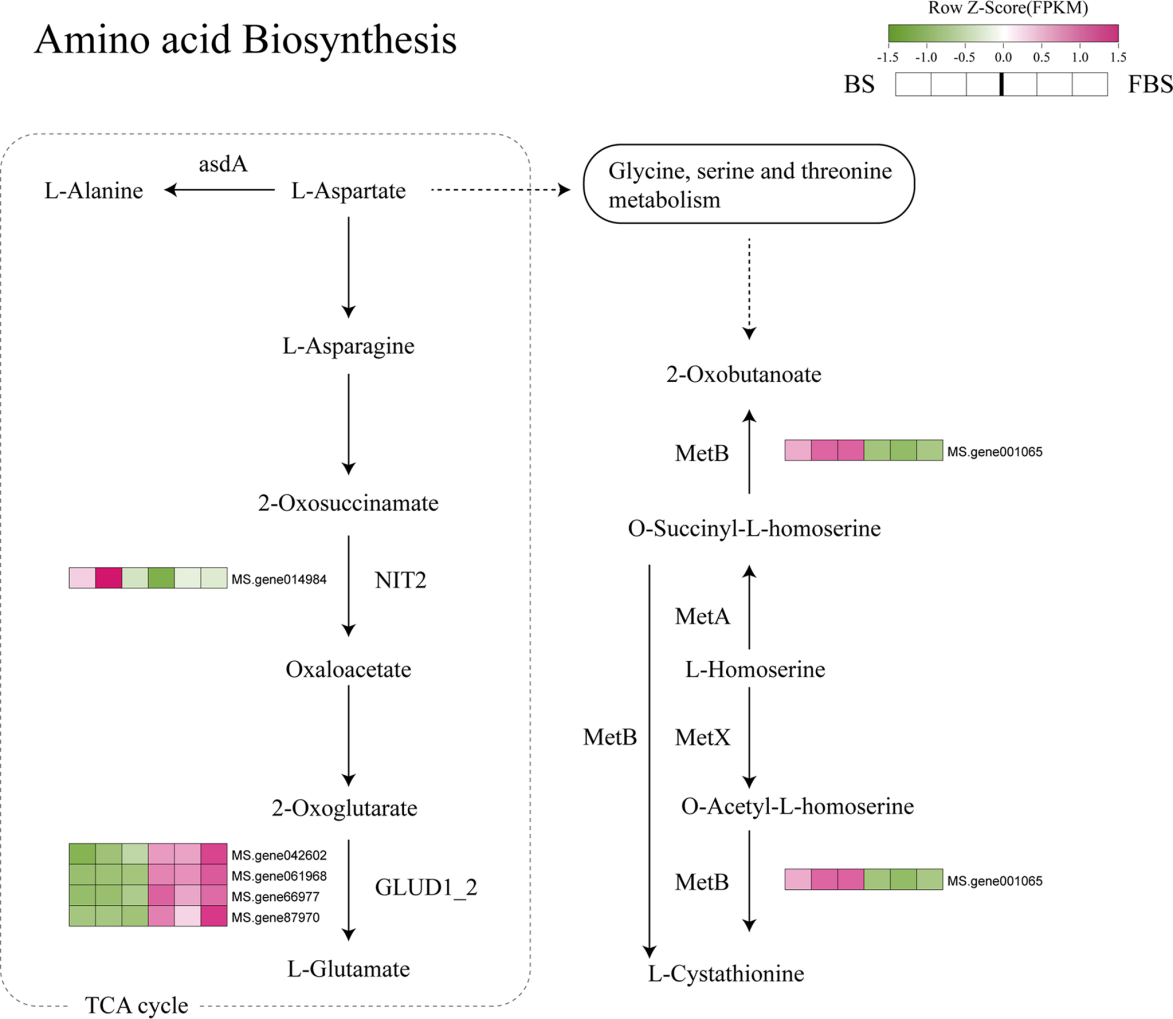
Amino acid biosynthesis pathway highlighting differentially expressed genes.

Based on our results describing changes in metabolite content, and transcription and translation of key genes, we have proposed some transcriptional regulatory relationships between candidate transcription factors and genes in the synthetic pathways of chlorophyll, amino acids, and flavonoids in alfalfa leaves (**Figures 5**–**7**). The *Hem* family genes regulate and catalyze many steps in the chlorophyll metabolism pathway, especially *HemA*, *HemL*, *HemE*, *HemY*, and *HemH*. From BS to FBS, some members of the *Hem* family are upregulated whereas other chlorophyll biosynthetic genes were downregulated, such as *ChIH*, *POR*, *DVR*, *CAO*, *ChlG*, and *NOL* (**Figure 5**).

CYP75B1 family genes participate in the flavonoid metabolic pathway. Indeed, among the up-regulated genes from BS to FBS are several genes belonging to this family, as well as many of the chalcone synthase gene family (**Figure 6**).

For the amino acid metabolism pathway, we found that *NIT2* and *metB* genes were down-regulated at FBS compared with BS, whereas *Glutamate dehydrogenase 1 (Glud1)* family genes were upregulated (**Figure 7**).

### Coexpression network analysis

**Figure 8:**
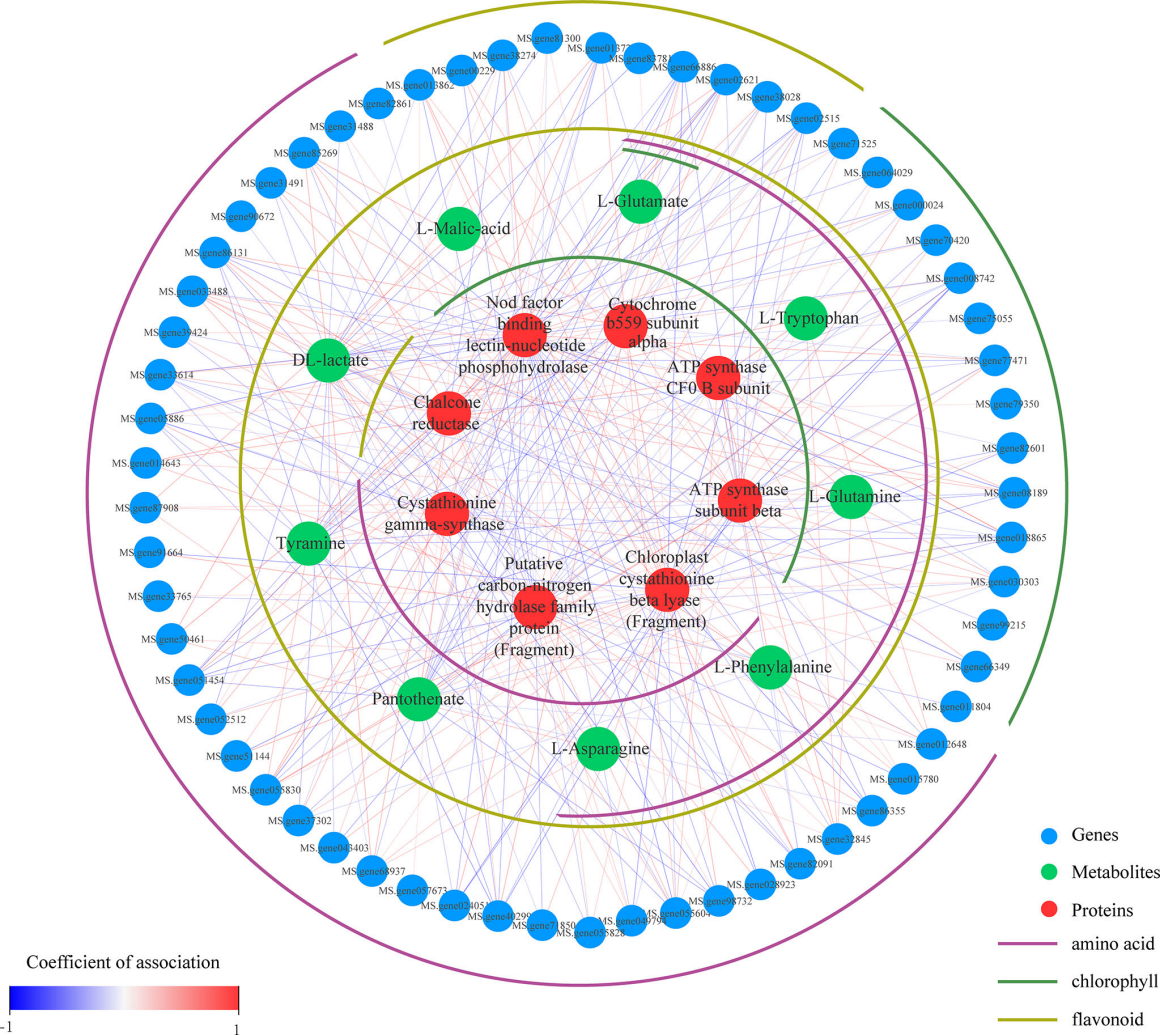
Network diagram of alfalfa metabolomic, proteomic, and transcriptomic analysis.

The genes, proteins, and metabolites of the chlorophyll, flavonoids, and amino acids coexpression network reveals 78 nodes and 327 edges (**Figure 8**). Seven encoded Hub proteins and three Hub metabolites were identified according to their margin >20. In this analysis, the interaction between the carbon-nitrogen hydrolase family protein (fragment) and dl-lactate was the strongest. Interestingly, there were relatively more negative correlation nodes between the metabolism of these hubs and proteins.

## Discussion

### Molecular regulation of chlorophyll biosynthesis in alfalfa leaves during flowering

Photosynthesis requires chlorophyll, which absorbs and converts solar energy for cell electron transfer and energy conversion (Zhang et al., 2021). The content of chlorophyll in alfalfa leaves affects not only yield and appearance, but also its nutritional quality (Saric-Krsmanovic et al., 2018). Our study indicates that there are some differences in metabolite accumulation between BS and FBS, especially chlorophyll that decreases significantly. Studies have shown that alfalfa forage protein significantly correlates with chlorophyll A and chlorophyll A/B, with chlorophyll A/B being the main factor affecting protein synthesis, followed by chlorophyll A (Liu, 2018). Therefore, the decrease in chlorophyll content may be one of the reasons for the decrease of protein content at different developmental stages.

The content of chlorophyll at BS was higher than at FBS. During early vegetative growth, alfalfa accumulates more nutrients and chlorophyll whereas during reproductive growth, the plants consume more nutrients than produced by photosynthesis while the chlorophyll content gradually declines. In contrast, Yu et al. (2017) found that chlorophyll content increased with leaf age. Chen et al. (2010) found that chlorophyll content differed during rice growth, peaking during heading and filling stages, and being low at seedling and tillering stages. Our divergent results may be due to differences between species, varieties, or environmental factors; or due to the fact that our study focused only on two developmental stages, close together but relevant for forage nutrition.

In our previous research, we found a direct correlation between chlorophyll and protein (Fan et al., 2018), but the specific regulatory mechanism was not then explained. Chlorophyll synthesis is triggered by the conversion of glutamate to 5-aminolevulinic acid (ALA). l-Glutamate is a precursor for chlorophyll synthesis, and an increased l-glutamate content in plants is beneficial for chlorophyll synthesis. After spraying glutamate on citrus leaves, the chlorophyll content was significantly higher in the treatment group (Wu et al., 2018). In another experiment, l-glutamate was added to the sugar-free medium of small plants (Gou et al., 2020), which increased the dry weight, chlorophyll content, and net photosynthetic rate in these plants. In alfalfa leaves, our metabolomic results revealed that l-glutamate content at FBS was significantly lower than at BS, in line with our previous experimental results.

ALA is a key precursor of chlorophyll biosynthesis catalyzed by glutamyl-tRNA synthetase, glutamyl-tRNA reductase (Glu TR), and glutamine-1-hemicaldehyde-aminotransferase. Glu TR, encoded by *HemA*, is the central controller of chlorophyll biosynthesis (Zeng et al., 2020; Luo et al., 2021). Other involved catalytic enzymes are bile pigment deaminase, urinary porphyrin synthase, urinary porphyrin iii decarboxylase, fecal porphyrin oxidation decarboxylase, and porphyrin oxidase (Zhang et al., 2010), encoded by *HemC*, *HemD*, *HemE*, *HemF*, and *HemG*, respectively. *HemA* is a gene family made up of multiple members, the number of which may vary from plant to plant. The model plant *Arabidopsis thaliana* has five *HemA* members, while cucumber has only two genes(Zhang et al., 2010). Here, we found 10 genes, including *HemA*, *HemB*, *HemC*, *HemD*, *HemE*, *HemF*, *HemH*, *HemY*, *HemL*, and *HemN*, five of which (*HemA*, *HemL*, *HemE*, *HemY*, and *HemH*) play decisive roles in the metabolism of l-glutamate to protoheme. To the best of our knowledge, this study has clarified the metabolic regulation pathway of *Hem* family genes in alfalfa for the first time.

A reduction in expression of *Hem* genes can lead to a decrease, or even loss, of enzyme activity and a corresponding decrease in chlorophyll production. Indeed, when *HemA* of *S. cerevisiae* was ectopically expressed in tobacco, transgenic plants oversynthesized ALA under light conditions and exhibited significantly enhanced leaf photosynthesis (Zhang et al., 2021). Li et al. (2020) showed that, in Arabidopsis, *HemA1* was induced by light, and its expression level changed with light quality (red, far-red, blue, and white light). Our results are consistent with previous studies, showing that the decrease in l-glutamate and the down-regulation of *HemA* expression may result in the inhibition of chlorophyll biosynthesis precursors, and ultimately lead to the decrease in leaf chlorophyll content.

### Molecular regulation of flavonoid biosynthesis in alfalfa leaves during flowering

The structurally diverse flavonoid metabolites contribute to various physiological plant processes, including biotic and abiotic stress response, nodule organogenesis (in legumes), fertility, pigmentation, and developmental regulation (Wang et al., 2020; Marco et al., 2021). Flavonoids also regulate root growth *via* reactive oxygen species (ROS) (Maloney et al., 2014) and the stomatal opening of guard cells (Watkins et al., 2017). Moderate salt stress in crops and medicinal plants can induce flavonoid accumulation to improve product quality (Lim et al., 2012). Flavonoids also help to resist infection by fungi and bacteria (Zhou et al., 2016).

Our study indicates that the flavonoid content in alfalfa leaves at FBS was significantly lower than at BS. This study provides the first report on how flavonoid content changes in alfalfa leaves during plant maturation, and indicates that flavonoid content is closely related to, and changes during, plant development. Similarly, in stevia, the accumulation of flavonoids is closely related to the growth stage, and its content show a dynamic trend of increasing (first 70 days), decreasing (day 70–94), and the remaining stable (Zhou et al., 2016).

Flavonoids in alfalfa mainly comprise apigenin, digloflavone, kainic acid, quercetin, and myricetin, among which the first two are the most common (Lui et al., 2020).

At present, *CYP75* family genes have attracted much attention, but there is no relevant report about *CYP75* in alfalfa. Relevant studies have shown that flavonoid synthesis in grapes is regulated by *CYP75* family genes, and the flavonoid content at the budding stage is significantly higher than that at flowering (Castellarin et al., 2006). Consistent with these results, this study has shown that *CYP75B1* plays a key role in the transformation of cinnamoyl-CoA into luteolin, apigenin and butin, especially in the transformation of liquintigenin to butin. Five genes (*Ms. Gene000023*, *Ms. Gene000024*, *Ms. Gene94506*, *Ms. Gene97810*, and *Ms. Gene97813*) were up-regulated, indicating that these genes play key roles in the transformation of these metabolites. However, their specific mechanisms of regulation require further elucidation.

### Molecular regulation of amino acid biosynthesis in alfalfa leaves during flowering

Alfalfa has a high total amino acid content, one of the important indices of plant quality (Liu et al., 2022). Studies have shown that the amino acid content is positively correlated with the crude protein content during growth, and that, with time, amino acid content decreases significantly (Liu, 2018). This observation is consistent with results from our proteomics analyses.

Amino acids are essential molecules that form proteins and contribute to the nitrogen cycle (Yu et al., 2020), and are also the precursors of many metabolites (Dinkeloo et al., 2018) such as the phytohormones auxin and ethylene (Du et al., 2022). Amino acid content and composition varies between tissues and cell types, and across different developmental stages (Kishor et al., 2015). To our knowledge, amino acid content, biosynthesis, and regulation in alfalfa leaves during development have not been investigated.

Increases in amino acid content may be due to increased amino acid biosynthesis or protein degradation; decreased glycolysis; or the metabolism of other nitrogen-containing compounds, including chlorophyll, purines, nucleotides, and alkaloids (Batista-Silva et al., 2019; Zhao et al., 2019). Here, we found that most DEGs between BS and FBS were involved in the glycolysis-TCA pathway, which suggests that decreased amino acid content in mature alfalfa leaves may be due to alterations in carbohydrate metabolism.

One member of the *NIT2* family was significantly down-regulated. Wang et al. (2010) described a similar phenomenon in chlorotic leaves of *Malus domestica* (apple), in which amino acid content was significantly decreased due to depressed glycolysis and TCA cycle activity. While *NIT2* is downregulated in the glutamate pathway, our results also showed that *MetB* family genes, participating in the metabolism of glycine to 2-oxobutanoate and the transformation and synthesis of l-cystathionine to 2-oxobutanoate, were significantly down-regulated. At present, there is no report on *MetB* in amino acid metabolism and synthesis, and its gene function needs to be further studied.

The glycolysis-TCA cycle synthesizes fundamental metabolites, such as amino acids and the reducing agent NADH, and provides energy for plant growth and development (Xiong et al., 2021). Phosphoenolpyruvate (PEP), an important intermediate in the glycolysis-TCA cycle, is also a precursor for amino acid biosynthesis, particularly aromatic amino acids (Li et al., 2020). Higher expression of glycolysis-TCA–related enzymes could increase amino acid synthesis (Katsuki et al., 2020). For example, wild ginseng has a higher amino acid content and higher expression of glycolysis-TCA–related enzymes than cultivated ginseng (Sun et al., 2016). TCA cycle intermediates have also been closely correlated with amino acid pools in tobacco (Zhao et al., 2016).

Results from this study clearly show that both glycolysis and the TCA cycle are downregulated in alfalfa leaves at budding compared with flowering, indicated by lower expression of several genes encoding key enzymes (e.g., *NIT2*, *metB*) and the decreased abundance of carbohydrate metabolism intermediates. These data provide the first description of amino acid regulation during alfalfa leaf development and provide the basis for further study to explore the precise roles of enzymes such as NIT2 and metB for use as precision tools to improve alfalfa leaf quality.

## Conclusion

We have analyzed the differences in chlorophyll, amino acid, and flavonoid content in alfalfa leaves at transcriptional, translational, and metabolic levels between two different developmental stages, the budding and the flowering stages. We found that as alfalfa matures, the content of chlorophyll, amino acids, and flavonoids significantly decreases. We identified ten *Hem* family genes present in alfalfa, which are involved in the l-glutamate regulatory and chlorophyll biosynthetic pathways. From BS to FBS, we observed a decrease in l-glutamate content and *HemA* expression in alfalfa leaves, which may inhibit the production of chlorophyll synthesis precursor and eventually lead to the observed decrease in leaf chlorophyll content. Expression of the key gene family *CYP75B1* decreased, resulting in a decline in flavonoids, such as apigenin and luteolin. In mature alfalfa leave, the amino acid synthesis genes *NIT2* and *metB* decreased, whereas *Glud1* increased, expression. Most DEGs were involved in the glycolysis-TCA pathway, causing a reduction in amino acid content and therefore nutritional quality. We have described some of the molecular mechanisms underpinning the nutritional value of alfalfa, providing a theoretical basis to guide future improvements in high-quality alfalfa hay production.

## Data availability statement

The data presented in the study are deposited in the NCBI repository, accession number PRJNA644634.

## Author contributions

YL and WF conducted the experiments, analyzed the data, and wrote the manuscript. QC, LZ, TC, XJ, and QY provided technical support, and revised the manuscript critically. QS and ZW provided the experimental materials. KJ conceived the experiment and manuscript revision. All authors contributed to the article and approved the final manuscript for submission.

## Funding

This work was supported by the Fundamental Research Funds of Chinese Academy of Agriculture (Grant No. 1610332022014), Inner Mongolia Autonomous Region science and technology planning project (Grant No. 2022YFHH0046), Inner Mongolia Natural Science Foundation project (Grant No2022LHQN3003) and Guizhou University Cultivation Project (Guida Renji Hezi [2020]9).

## Acknowledgments

We would like to thank all colleagues and friends who have contributed to this study.

## Conflict of interest

The authors declare that the research was conducted in the absence of any commercial or financial relationships that could be construed as a potential conflict of interest.

## Publisher’s note

All claims expressed in this article are solely those of the authors and do not necessarily represent those of their affiliated organizations, or those of the publisher, the editors and the reviewers. Any product that may be evaluated in this article, or claim that may be made by its manufacturer, is not guaranteed or endorsed by the publisher.

## References

[B1] Batista-Silva W. Heinemann B. Rugen N. Nunes-Nesi A. Araújo W. L. Braun H. P.. (2019). The role of amino acid metabolism during abiotic stress release. Plant Cell Environ. 42 (5), 1630–1644. doi: 10.1111/pce.13518 30632176

[B2] Bird P. R. Moir R. J. (1972). Sulphur metabolism and excretion studies in ruminants. 8. methionine degradation and utilization in sheep when infused into the rumen or abomasum. Aust. J. Biol. Sci. 25, 835–848. doi: 10.1071/bi9731429 5086943

[B3] Castellarin S. D. Di Gaspero G. Marconi R. Nonis A. Peterlunger E. Paillard S.. (2006). Colour variation in red grapevines (Vitis vinifera l.): genomic organisation, expression of flavonoid 3'-hydroxylase, flavonoid 3',5'-hydroxylase genes and related metabolite profiling of red cyanidin-/blue delphinidin-based anthocyanins in berry skin. BMC Genomics 7 (1), 12. doi: 10.1186/1471-2164-7-12 16433923 PMC1403756

[B4] Chen X. L. Chen C. Zhou L. (2010). Determination and correlativity analysis of chlorophyll content at different developmental stages in rice. Modern Agric. Sci. Technology. 17), 42–44,52. doi: 10.3969/j.issn.1007-5739.2010.17.017

[B5] Dantas R. Rodrigo S. Constantin P. Barbosa R. Anderson L. Soares R.. (2020). Biosynthesis and metabolic actions of simple phenolic acids in plants. Phytochem. Rev. 19, 865–906. doi: 10.1007/s11101-020-09689-2

[B6] Dinkeloo K. Boyd S. Pilot G. (2018). Update on amino acid transporter functions and on possible amino acid sensing mechanisms in plants. Semin. Cell Dev. Biol. 74, 105.133. doi: 10.1016/j.semcdb.2017.07.010 28705659

[B7] Dong S. Liu T. Dong M. (2018). Determination of 18 kinds of amino acids in fresh tea leaves by hplc coupled with pre-column derivatization. Asian Agric. Res. 10 (02), 59–62+67.

[B8] Dong S. W. Sang L. J. Xie H. L. Chai M. F. Wang Z. Y. (2021). Comparative transcriptome analysis of salt stress-induced leaf senescence in medicago truncatula. Front. Plant Science. 12. doi: 10.3389/fpls.2021.666660 PMC8299074 34305965

[B9] Du Z. K. Lin W. D. Zhu J. X. Li J. M. (2022). Amino acids profiling and transcriptomic data integration demonstrates the dynamic regulation of amino acids synthesis in the leaves of cyclocarya paliurus. PeerJ. 10, e13689. doi: 10.7717/peerj.13689 35811808 PMC9266588

[B10] Fan W. Q. Ge G. T. Liu Y. H. Wang W. Liu L. Y. Jia Y. S. (2018). Proteomics integrated with metabolomics: analysis of the internal causes of nutrient changes in alfalfa at different growth stages. BMC Plant Biol. 18 (1), 78. doi: 10.1186/s12870-018-1291-8 29728056 PMC5935980

[B11] Galili G. Amir R. Fernie A. R. (2016). The regulation of essential amino acid synthesis and accumulation in plants. Annu. Rev. Plant Biol. 67, 8.1–8.26. doi: 10.1146/annurev-arplant-043015-112213 26735064

[B12] Gao J. Wang H. Yuan Q. Yue F. (2018). Structure and function of the photosystem supercomplexes. Front. Plant Science. 9. doi: 10.3389/fpls.2018.00357 PMC5869908 29616068

[B13] Goawska S. Ukasik I. Kapusta T. Janda B. (2010). Analysis of flavonoids content in alfalfa. Ecol. Chem. Engineering 17 (2–3), 261–267.

[B14] Gou T. Y. Yang L. Hu W. X. Chen X. H. Zhu Y. X. Guo J.. (2020). Silicon improves the growth of cucumber under excess nitrate stress by enhancing nitrogen assimilation and chlorophyll synthesis. Plant Physiol. Biochem. 152, 53–56. doi: 10.1016/j.plaphy.2020.04.031 32388420

[B15] Guo H. M. Zhu H. S. Guo J. X.. (2020). Comparative study on extraction and detection methods of total flavonoids from Alfalfa at different growth stages. Chinese Journal of Grassland 42 (03), 141–146. doi: 10.16742/j.zgcdxb.20190270

[B16] Harmut A. (1987). Chlorophylls and carotenoids: Pigments of photosynthetic membranes. Methods Enzymology. 148, 350–383. doi: 10.1016/0076-6879(87)48036-1

[B17] Jing D. Chen W. Hu R. Zhang Y. Liang G. (2020). An integrative analysis of transcriptome, proteome and hormones reveals key differentially expressed genes and metabolic pathways involved in flower development in loquat. Int. J. Mol. Sci. 21 (14), 5107. doi: 10.3390/ijms21145107 32698310 PMC7404296

[B18] Jing C. L. Dong X. F. Tong J. M. (2015). Optimization of ultrasonic-assisted extraction of flavonoid compounds and antioxidants from alfalfa using response surface method. Molecules 20 (9), 15550–15571. doi: 10.3390/molecules200915550 26343617 PMC6332291

[B19] Katsuki M. Daisuke S. Shunsuke K. (2020). Optimal ratio of carbon flux between glycolysis and the pentose phosphate pathway for amino acid accumulation in corynebacterium glutamicum. ACS Synthetic Biol. 9 (7), 1615–1622. doi: 10.1021/acssynbio.0c00181 32602337

[B20] Kishor P. B. K. HimaKumari P. Sunita M. S. L. Sreenivasulu N. (2015). Role of proline in cell wall synthesis and plant development and its implications in plant ontogeny. Front. Plant Science. 6. doi: 10.3389/fpls.2015.00544 PMC4507145 26257754

[B21] Less H. Galili G. (2009). Coordinations between gene modules control the operation of plant amino acid metabolic networks. BMC Syst. Biol. 3, 14. doi: 10.1186/1752-0509-3-14 19171064 PMC2646696

[B22] Li N. Liu Y. Liang Z. Lou Y. Wang G. (2020). Influence of fuel oil on platymonas helgolandica: an acute toxicity evaluation to amino acids. Environ. Pollution. 271, 116226. doi: 10.1016/j.envpol.2020.116226 33360349

[B23] Lim J. Park K. Kim B. (2012). Effect of salinity stress on phenolic compounds and carotenoids in buckwheat sprout. Food Chem. 135 (3), 1065–1070. doi: 10.1016G/j.foodchem.2012.05.068 22953825

[B24] Liu L. Y. (2018). Effects of environmental factors on nutrients in alfalfa drying process and field regulation strategies (Inner Mongolia Agricultural University).

[B25] Liu L. Y. Jia Y. S. Wang Z. J. (2022). Study on main environmental factors affecting natural drying of alfalfa. J. Grass Industry. 31 (2), 121–132. doi: 10.11686/cyxb2020529

[B26] Lui A. C. W. Lam P. Y. Kwun H. C. Wang L. X. Tobimatsu Y. Lo C. (2020). Convergent recruitment of 5'-hydroxylase activities by CYP75B flavonoid b-ring hydroxylases for tricin biosynthesis in medicago legumes. New Phytol 228 (1), 268–284. doi: 10.1111/nph.16498 32083753

[B27] Luo J. Wang H. Chen S. Ren S. (2021). CmNAC73 mediates the formation of chrysanthemum green by directly activating the expression of chlorophyll biosynthesis genes HEMA1 and CRD1. Genes 12 (5), 704. doi: 10.3390/genes12050704 34066887 PMC8151904

[B28] Maloney G. Dinapoli K. Muday G. (2014). The anthocyanin reduced tomato mutant demonstrates the role of flavonols in tomato lateral root and root hair development. Plant Physiol. 166 (2), 614–631. doi: 10.1104/pp.114.240507 25006027 PMC4213093

[B29] Marco A. Mariana B. Natália G. Maycon A. Cleverson R. Jaquelyne P.. (2021). Physiological impact of flavonoids on nodulation and ureide metabolism in legume plants. Plant Physiol. Biochem. 166 (8), 512–521. doi: 10.1016/j.plaphy.2021.06.007 34171572

[B30] Ouyang K. H. Xu M. S. Jiang Y. Wang W. J. (2016). Effects of alfalfa flavonoids on broiler performance, meat quality and gene expression. Can. J. Anim. Science. 96 (3), 332–341. doi: 10.1139/CJAS-2015-0132

[B31] Popovic S. Stjepanovic M. Grljusic S. Cupic T. Tucak M. (2001). Protein and fiber contents in alfalfa leaves and stems. Quality in Lucerne and Medics for Animal Production (Eds.: Delgado I. Lloveras J. 45, 215–218.

[B32] Ren Y. Pan J. Zhang Z. Zhao J. Hu G. (2020). Identification of an up-accumulated polyamine oxidase 2 in pollen of self-incompatible 'wuzishatangju' mandarin using comparative proteomic analysis. Scientia Horticulturae. 266, 109279. doi: 10.1016/j.scienta.2020.109279

[B33] Saric-Krsmanovic M. Bozic D. Radivojevic L. Gajic Umiljendic J. Vrbnicanin S. (2018). Impact of field dodder (cuscuta campestris yunk.) on chlorophyll fluorescence and chlorophyll content of alfalfa and sugar beet plants. Russian J. Plant、Physiology. 65 (5), 726–731. doi: 10.1134/S102144371805014X

[B34] Schmied J. Hedtke B. Grimm B. (2011). Overexpression of hema1 encoding glutamyl-trna reductase. J. Plant Physiol. 168 (12), 1372–1379. doi: 10.1016/j.jplph.2010.12.010 21272955

[B35] Shalygo N. Czarnecki O. Peter E. Grimm B. (2009). Expression of chlorophyll synthase is also involved in feedback-control of chlorophyll biosynthesis. Plant Mol. Biol. 71 (4), 425–436. doi: 10.1007/s11103-009-9532-8 19680747

[B36] Stenbaek A. Jensen P. E. (1995). Chlorophyll biosynthesis. Phytochemistry 7 (7), 1039–1057. doi: 10.2307/3870056

[B37] Sun H. Liu F. B. Sun L. W. (2016). Proteomic analysis of amino acid metabolism differences between wild and cultivated panax ginseng. J. Ginseng Res. 40 (2), 113–120. doi: 10.1016/j.jgr.2015.06.001 27158231 PMC4845045

[B38] Tong Z. Y. Xie C. Ma L. Liu L. P. Jin Y. S. Dong J. L.. (2014). Co-Expression of bacterial aspartate kinase and adenylylsulfate reductase genes substantially increases sulfur amino acid levels in transgenic alfalfa (medicago sativa l.). PloS One 9 (2), e88310. doi: 10.1371/journal.pone.0088310 24520364 PMC3919742

[B39] Wang F. Ge S. F. Xu X. X. Xin Y. X. Zhang D. X. (2021). Multiomics analysis reveals new insights into the apple fruit quality decline under high nitrogen conditions. J. Agric. Food Chem. 69 (19), 5559–5572. doi: 10.1021/acs.jafc.1c01548 33945277

[B40] Wang Z. H. Hong X. Hu K. K. Wang Y. Wang X. X. Du S. Y.. (2017). Impaired magnesium protoporphyrin ix methyltransferase (chlm) impedes chlorophyll synthesis and plant growth in rice. Front. Plant Sci. 8, 1694. doi: 10.3389/fpls.2017.01694 29033966 PMC5626950

[B41] Wang H. J. Liu S. H. Wang T. L. Liu H. W. Xu X. H. Chen K. S.. (2020). The moss flavone synthase I positively regulates the tolerance of plants to drought stress and UV-b radiation. Plant Science. 298, 110591. doi: 10.1016/j.plantsci.2020.110591 32771149

[B42] Wang H. Ma F. Cheng L. (2010). Metabolism of organic acids, nitrogen and amino acids in chlorotic leaves of ‘Honeycrisp’ apple (Malus domestica borkh) with excessive accumulation of carbohydrates. Planta. 232, 511–522. doi: 10.1007/s00425-010-1194-x 20490541

[B43] Wang Z. Q. Shi H. R. Yu S. F. Zhou W. L. Li J. Liu S. H.. (2019). Comprehensive transcriptomics, proteomics, and metabolomics analyses of the mechanisms regulating tiller production in low-tillering wheat. Theor. Appl. Genet. 132, 2181–2193. doi: 10.1007/s00122-019-03345-w 31020386

[B44] Watkins J. Chapman J. Muday G. (2017). Abscisic acid- induced reactive oxygen species are modulated by flavonols to control stomata aperture. Plant Physiol. 175 (4), 1807–1825. doi: 10.1104/pp.17.01010 29051198 PMC5717730

[B45] Wisniewski J. R. Zougman A. Nagaraj N. Mann M. (2009). Universal sample preparation method for proteome analysis. Nat. Methods 6, 359–362. doi: 10.1038/nmeth.1322 19377485

[B46] Wu Y. Xin J. Liao W. Hu L. Dawuda M. M. Zhao X.. (2018). 5-aminolevulinic acid (ala) alleviated salinity stress in cucumber seedlings by enhancing chlorophyll synthesis pathway. Front. Plant Science. 9. doi: 10.3389/fpls.2018.00635 PMC5962685 29868088

[B47] Xiong Y. Qu. Y. Han H. (2021). Unraveling physiological and metabolomic responses involved in phlox subulata l. tolerance to drought stress. Plant Mol. Biol. Rep. 39, 98–111. doi: 10.1007/s11105-020-01238-7

[B48] Xu B. Wu R. L. Shi F. L. Gao C. Wang J. (2022). Transcriptome profiling of flower buds of male-sterile lines provides new insights into male sterility mechanism in alfalfa. BMC Plant Biology 22 (1), 1–13. doi: 10.1186/s12870-022-03581-1 35428186 PMC9013074

[B49] Yu W. X. Yang Q. P. Huang S. M. Guo Z. W. Yu D. Q. (2017). Analysis on the main influencing factors of chlorophyll value in leaves of phyllostachys prominens. J. Zhejiang Forestry Sci. Technology. 2), 36–39. doi: 10.3969/j.issn.1001-3776.2014.02.009

[B50] Zeng Z. Q. Lin T. Z. Zhao J. Y. Zheng T. H. (2020). The OsHemA gene encoding glutamyl-TrNA reductase (GluTR) is critical for chlorophyll biosynthesis in rice (oryza sativa). J. Integr. Agriculture. 19 (3), 12. doi: 10.1016/s2095-3119(19)62710-3

[B51] Zhang J. L. Li X. G. Xu X. H. Chen H. P. Li Y. L. Guy R. D. (2021). Leaf morphology, photosynthesis and pigments change with age and sunlight in savin juniper. Plant Biol. 23 (6), 1097–1108. doi: 10.1111/plb.13256 33756015

[B52] Zhang C. H. Zhang H. Y. Yu X. Z. (2010). Effects of selenium on photosynthetic characteristics and chlorophyll fluorescence parameters of tomato seedling leaves under low temperature stress. Chin. Agric. Sci. Bulletin. 26 (5), 152–157.

[B53] Zhan J. S. Liu M. M. Su X. S. Zhan K. Zhang C. G. Zhao G. Q. (2017). Effects of alfalfa flavonoids on the production performance, immune system, and ruminal fermentation of dairy cows. Asian-Australasian J. Anim. Sci. 30 (10), 1416–1424. doi: 10.5713/ajas.16.0579 PMC5582326 28423878

[B54] Zhao Y. Xu F. L. Liu J. Guan F. C. Quan H. Meng F. J. (2019). The adaptation strategies of herpetospermum pedunculosum (Ser.) baill at altitude gradient of the Tibetan plateau by physiological and metabolomic methods. BMC Genomics 20, 451. doi: 10.1186/s12864-019-5778-y 31159723 PMC6547600

[B55] Zhao J. Zhao Y. Hu C. Zhao C. Zhang J. Li L. (2016). Metabolic profiling with gas chromatography-mass spectrometry and capillary electrophoresis-mass spectrometry reveals the carbon-nitrogen status of tobacco leaves across different planting areas. J. Proteome Res. 15, 468–476 468. doi: 10.1021/acs.jproteome.5b00807 26784525

[B56] Zhou X. He J. Wang T. (2016). Combined effects of blue and ultraviolet lights on the accumulation of flavonoids in tartary buckwheat sprouts. Polish J. Food Nutr. Sci. 66 (2), 93–98. doi: 10.1515/pjfns-2015-0042

