# Supplementary material for: Multi-omics analyses reveal new insights into nutritional quality changes of alfalfa leaves during the flowering period
Source: Front Plant Sci. 2022 Nov 30;13:995031. doi: 10.3389/fpls.2022.995031 (PMC9748345; doi:10.3389/fpls.2022.995031)
Supplement: Supplementary file 1 [file DataSheet_1.docx]

**Supplementary Material**

**
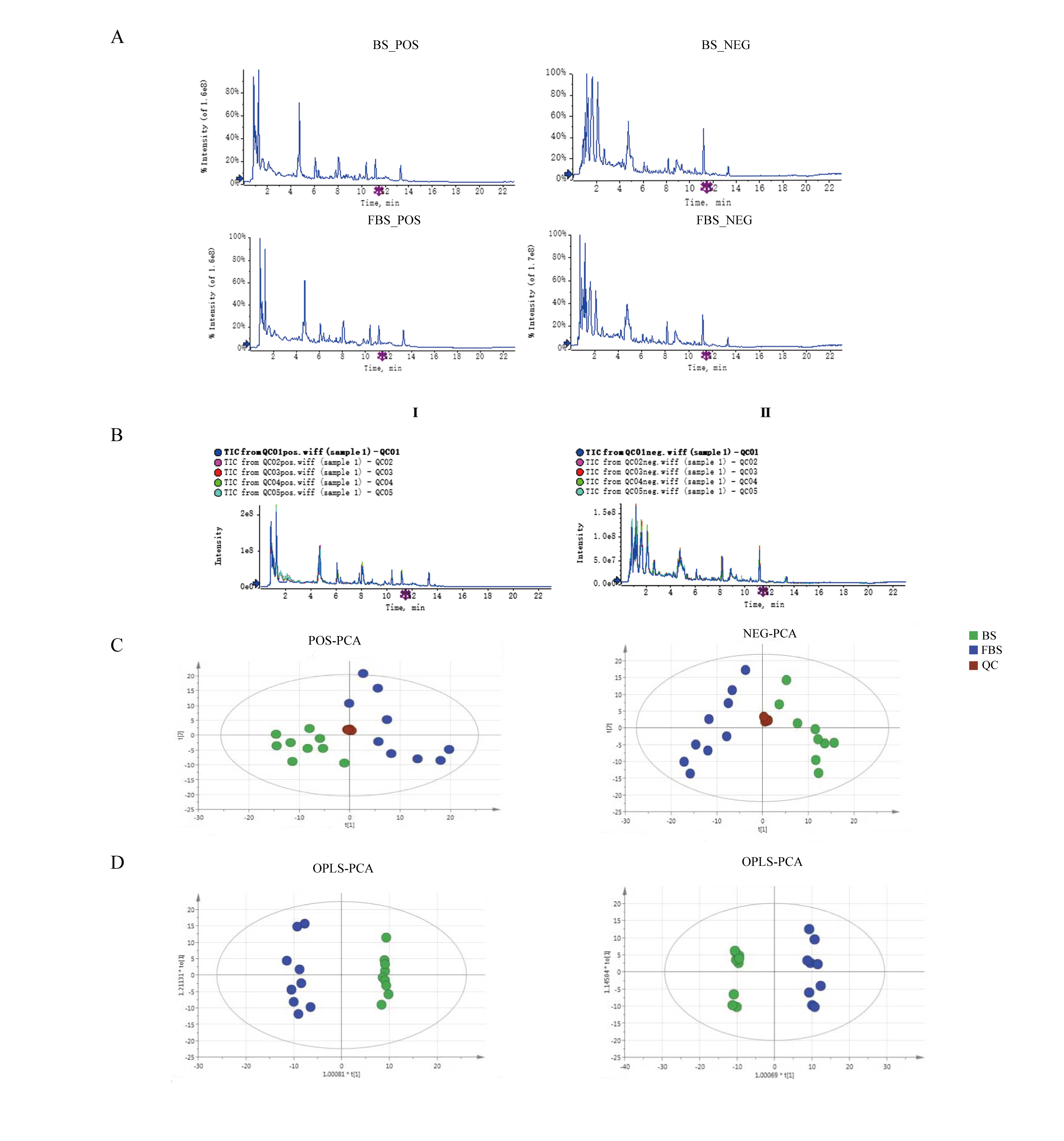
**

**Supplementary Figure 1**. (A) TIC overlap pattern of positive and negative ion modes. (B) TIC pattern of positive and negative ion patterns. (C) PCA of positive and negative ion modes. (D) OPLS-DA of positive and negative ion modes.

|  | **Sample** | | | | | |
| --- | --- | --- | --- | --- | --- | --- |
|  | **BS1** | **BS2** | **BS3** | **FBS1** | **FBS2** | **FBS3** |
| Raw Reads | 42,519,082 | 41,751,628 | 42,137,334 | 41,107,062 | 43,027,628 | 41,975,256 |
| Raw Bases (kb) | 6,377,862.3 | 6,262,744.2 | 6,320,600.1 | 6,166,059.3 | 6,454,144.2 | 6,296,288.4 |
| Clean Reads | 41,283,346 | 40,427,228 | 41,235,202 | 40,300,868 | 41,934,052 | 40,741,398 |
| Clean Reads Rate (%) | 97.09 | 96.83 | 97.86 | 98.04 | 97.46 | 97.06 |
| Clean Bases (kb) | 6,192,501.9 | 6,064,084.2 | 6,185,280.3 | 6,045,130.2 | 6,290,107.8 | 6,111,209.7 |
| Raw Q30 Rate (%) | 92.22 | 92.29 | 91.3 | 91.61 | 92.51 | 92.18 |
| Clean Q30 Rate (%) | 93.01 | 93.01 | 92.01 | 92.15 | 93.17 | 92.97 |
| Mapping Rate (%) | 90.12 | 88.45 | 89.76 | 88.67 | 89.92 | 89.35 |
| MultiMap Rate | 0.5134 | 0.4942 | 0.5067 | 0.5029 | 0.5093 | 0.5066 |

**Supplementary Table 1**. RNA-seq library read numbers and quality parameters.
